# The Effects of Tyrosine Hydroxylase Blockade in Mice Lacking the Norepinephrine Transporter (NET-KO Mice)

**DOI:** 10.3390/ijms27083656

**Published:** 2026-04-20

**Authors:** Zoia S. Fesenko, Anna B. Volnova, Evgeniya V. Efimova, Tatyana D. Sotnikova, Raul R. Gainetdinov

**Affiliations:** 1 Institute of Translational Biomedicine, St. Petersburg State University, Universitetskaya Nab. 7/9, 199034 St. Petersburg, Russia; 2 Center for Transgenesis and Genome Editing, St. Petersburg State University, Universitetskaya Nab. 7/9, 199034 St. Petersburg, Russia

**Keywords:** dopamine, norepinephrine, norepinephrine transporter (NET), NET-KO mice, Parkinson’s disease

## Abstract

In recent years, significant progress has been made in understanding that Parkinson’s disease (PD) is associated not only with the dopamine (DA) but also with the norepinephric (NE) system. In order to investigate the potential involvement of NE in the development of the early motor symptoms of PD, we studied the effects of reducing its levels in a norepinephrine transporter knockout mouse (NET-KO). Due to the absence of NET, all the norepinephrine needed must be synthesized de novo. NET-KO mice were injected intraperitoneally with α-methyl-p-tyrosine (AMPT), a blocker of tyrosine hydroxylase, to induce a hyponoradrenergic state. Changes in tissue NE content in the frontal cortex and DA content in the striatum were evaluated using HPLC. We also measured the motor activity parameters of NET-KO mice after AMPT injection. The hyponorepinephric state induced by AMPT administration in NET-KO mice did not lead to severe motor impairments, as occurs in PD models. However, NET-KO mice did exhibit abnormal hindlimb extension, which began three hours after AMPT administration. This symptom may be interpreted as an early symptom preceding PD. These results suggest that the potential involvement of different neurotransmitter systems in motor abnormalities relevant to Parkinson’s disease warrants further investigation.

## 1. Introduction

Parkinson’s disease (PD) is a progressive neurodegenerative disorder that affects multiple systems. It manifests with both motor and non-motor symptoms. Motor symptoms include tremors, rigidity, bradykinesia, and postural instability, while non-motor symptoms encompass autonomic dysfunction, sensory disturbances, sleep disorders, cognitive impairment, behavioral changes, dementia, and depression [1,2,3,4]. PD is characterized by the loss of dopamine (DA) neurons in the substantia nigra (SN) pars compacta, primarily due to the accumulation of Lewy bodies—aggregates of alpha-synuclein. By the time patients display visual symptoms of PD, DA levels drop by 70% [5,6,7,8,9]. Lewy bodies can be found not only in DA neurons but also in glutamatergic, norepinephrinergic, serotoninergic, histaminergic, and cholinergic neurons. Moreover, before significant degeneration occurs in the substantia nigra, damage to the anterior olfactory structures, motor nucleus of the vagus nerve, inferior raphe system, and locus coeruleus (LC) also takes place [10,11,12,13,14,15,16,17].

Research indicates that neuronal degeneration in the LC is as extensive as in the SN [16,18,19]. Postmortem studies reveal a 30–90% loss of norepinephrine-producing cells in the LC in PD patients, with the remaining cells often filled with Lewy bodies, indicating moderate to severe degeneration [3,20,21]. There is also some evidence suggesting that degeneration of norepinephrine-producing cells can occur even prior to degeneration of DA neurons [19,20,22,23]. Norepinephrine (NE) synthesis is significantly reduced throughout the brain in PD, with some regions having less than half of normal concentrations of NE. Several years prior to the detection of dopaminergic neuron degeneration, the cerebrospinal fluid showed decreased levels of dopamine-beta-hydroxylase, an enzyme responsible for converting dopamine into norepinephrine. Therefore, research indicates that the extent of neuronal degeneration in the LC is comparable in severity to that observed in the SN and begins a few years earlier [18,19,24].

Also, it was shown that NE may help protect DA neurons, and damage to the norepinephrine system could accelerate the progression of PD [25,26,27]. Noradrenaline is both an independent neurotrophic factor and a molecule that promotes the expression of brain-derived neurotrophic factor (BDNF), fibroblast growth factor-2 (FGF-2), Bcl-2, and nerve growth factor (NGF). Stimulation of adrenergic receptors promotes anti-inflammatory immune responses by reducing cytokine secretion and nitric oxide synthase (NOS) expression. Noradrenergic activity reduces the production of free radicals and, along with its active metabolites, prevents lipid peroxidation in cell membranes. Studies using 1-methyl-4-phenyl-1,2,3,6-tetrahydropyridine (MPTP)-induced PD models in mice and monkeys have shown that greater damage to the LC leads to increased DA neuron loss in the SN, resulting in more severe motor deficits [3,28,29,30]. And enhancing NE synthesis or knocking out the norepinephrine transporter (NET) gene reduces MPTP-induced neurodegeneration and motor impairments. In a rat model of PD with lipopolysaccharide (LPS)-induced inflammation, peripheral administration of atomoxetine, a NET blocker, decreased DA neuron damage, whereas administration of the DSP-4 toxin, which selectively destroys NE neurons, resulted in both motor impairments and DA cell loss [3]. The microinjections of transcription factors Phox2a/2b or their combination with Hand2 or Gata3 into the LC of a vesicular monoamine transporter-2 (VMAT2)-deficient transgenic mouse model, in which the endogenous expression of VMAT2 was stopped by almost 95%, increased the levels of dopamine-beta-hydroxylase (DBH) and tyrosine hydroxylase (TH) protein in the LC, frontal cortex, and hippocampus, TH in the striatum and the SN, as well as improved cognitive and motor function. Notably, these transcription factors also play a role in noradrenergic neuron embryonic development, and they are also present in adult brain noradrenergic neurons, supporting the expression of DBH and TH [31]. These findings suggest that norepinephrine system dysfunction contributes to a decline in DA levels and impaired motor function.

Studies with selective agonists and antagonists of α-adrenergic receptors have shown that the α2-adrenergic receptor may play a role in controlling motor activity. Antagonists of the α2-adrenergic receptor have been shown to improve motor symptoms in patients with PD and may also reduce levodopa-induced dyskinesias. Studies have shown that the beta-adrenergic antagonist nadolol reduces PD tremors. L-DOPS (droxidropa), an artificial NE precursor, improved gait and balance in patients [12,32,33,34,35,36,37]. However, the underlying physiological mechanisms behind the relationship between the norepinephrine system and motor dysfunction remain unclear.

For a long time, PD models have focused on the depletion of DA [38,39,40,41]. One of these models, showing the crucial role of DA in the motor manifestations of PD, was created using mice lacking a DA transporter (DAT-KO mice) [42]. DAT-KO mice are characterized by increased levels of extracellular dopamine in the striatum, leading to hyperactivity, stereotypical behavior, cognitive deficits, and other behavioral characteristics [42,43,44,45]. In 2005, a paper was published on the pharmacological blockade of the enzyme tyrosine hydroxylase, which limits the rate of dopamine synthesis, in DAT-KO mice using the compound α-methyl-p-tyrosine (AMPT)—DDD mice (dopamine deficiency in DAT-KO mice). As a result, DA levels in the brains of knocked-out animals, both extracellularly and in tissue, decreased to undetectable levels, causing a short-term manifestation of PD symptoms for about 16 h. The administration of AMPT to DAT-KO mice led to the development of a specific akinetic phenotype. Excessive rigidity and body tremors developed, and all manifestations reached their maximum within 30–60 min after injection. Further tests showed that akinesia was not associated with general sedation, namely, lack of movement control. DDD mice were able to swim for long periods, indicating the possibility of movement without sedation under certain conditions. The injections of L-DOPA (levodopa) + carbidopa effectively restored locomotion [46,47].

In wild-type mice (WT), AMPT does not affect motor function. WT mice do not show any signs of akinesia, rigidity, or body tremor. The DA concentration in the striatal tissue decreased by approximately 40%, while the extracellular level remained unchanged. The NE level in the frontal cortex decreased by approximately 35%, compared to normal levels. However, there was no significant difference between DAT-KO and WT mice in terms of NE reduction [46,47]. Due to the marked behavioral phenotype, the DDD model suggests that DA is essential for motor function. However, it does not address the question of whether PD motor manifestations are solely dependent on DA or if NE can also play a role.

Mice lacking the NE transporter were also created (NET-KO mice). NET-KO mice are viable, fertile, and exhibit reduced body temperature and body weight compared to controls, which is a similar feature of DAT-KO mice [42,48]. In NET-KO mice, the concentration of norepinephrine was reduced by more than half in the tissues of the prefrontal cortex, hippocampus, and cerebellum, and the rate of NE clearance was reduced six times compared to WT [48]. The disruption of the dynamics of NE reuptake and, consequently, the clearance of the extracellular space of released NE leads to an increase in the extracellular concentration of NE, which is supported by the findings from microdialysis and the specific binding of desipramine. The absence of the norepinephrine transporter also affects the dopamine system: in NET-KO mice, the rate of DA synthesis is reduced, as well as tissue concentrations of DA and its metabolites in the striatum [48]. A noteworthy distinction is the response to novelty in DAT-KO mice and NET-KO mice: mice with hyperdopaminergia (DAT-KO mice) show high motor responses to novelty, whereas mice with hypernorepinephria (NET-KO mice) exhibit reduced motor responses to novelty [45,48]. Apart from that, in the forced swimming test and the tail suspension test, NET-KO showed reduced immobilization time. This effect is similar to the action of tricyclic antidepressants (desipramine, bupropion), which target NET [48,49,50,51].

NE is synthesized from DA; therefore, TH is also involved in its synthesis. Acute pharmacological inhibition of TH with AMPT in NET-KO mice can result in a lack of NE, similar to a lack of DA in the DDD model. In this study, we investigated the effect of TH blockade with AMPT in norepinephrine transporter knockout mice (NDN mice—norepinephrine deficiency in NET-KO mice) to determine the possible NE involvement of motor disorders, in particular, for example, in the early development of motor symptoms in PD.

## 2. Results

**Figure 1:**
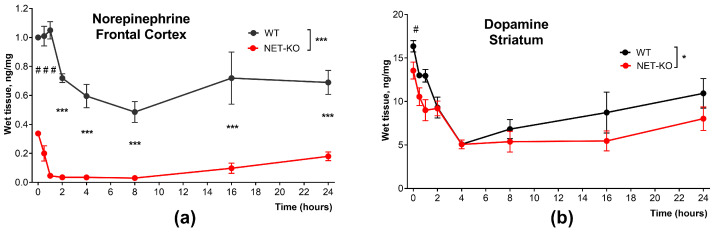
The effect of AMPT administration on the concentrations of (**a**) tissue norepinephrine in the frontal cortex and (**b**) tissue dopamine in the striatum in WT and NET-KO mice 0.5, 1, 2, 4, 8, 16, and 24 h after injection. AMPT 250 mg/kg IP, n = 5 per group per each time point. #—*p* < 0.05; ###—*p* < 0.001; two-tailed unpaired *t* test; *—*p* < 0.05; ***—*p* < 0.001; two-way ANOVA with Sidak’s multiple comparisons test.

First, we measured how the administration of AMPT influenced the concentration of NE and DA. Previously, it was shown that the content of norepinephrine in the tissues of mice with norepinephrine transporter knockout is significantly lower than in wild-type mice [48]. In our work, we also observed that before AMPT administration, NE levels were significantly lower in NET-KO mice compared to WT mice in the frontal cortex (two-tailed unpaired t test, *p* < 0.0001 for both structures) (Figure 1a). The administration of AMPT significantly reduced the concentration of NE in the tissue in both NET-KO and WT mice (Figure 1a). In WT mice, the concentration of NE started to decrease 2 h after injection and reached 50% in 4–8 h (*p* < 0.001; factor “time”; two-way ANOVA). That level of NE in WT gradually normalized. However, even after 24 h, it remained 70% of the intact level.

In NET-KO mice, administration of AMPT decreased the level of NE more rapidly and more drastically than in WT mice. One hour after injection in NET-KO mice, the level of NE in the frontal cortex was reduced almost to zero (Figure 1a). And that extremely low level remained for 8 h, and at 16 h it slowly started to grow. In NET-KO mice, 24 h after AMPT injection, the concentration of NE in the frontal cortex was less than 40% of the intact level.

Thus, significant differences were observed in the composition of samples from the frontal cortex (*p* < 0.001, factor “genotype”, two-way ANOVA) when comparing NET-KO and WT. Sidak’s multiple comparisons test also revealed significant differences at 2, 4, 8, 12, and 24 h after injection (*p* < 0.001).

The tissue content of DA in the striatum before AMPT administration was slightly lower in NET-KO mice compared to WT mice (two-tailed unpaired *t* test, *p* = 0.045; Figure 1b). Administration of AMPT led to a drop in the concentration of DA in the striatum tissue that started 30 min after injection and reached the lowest concentration by the fourth hour of the experiment (*p* < 0.0001; factor “time”; two-way ANOVA). Two hours after the administration of AMPT, the concentration of DA did not differ between NET-KO and WT mice. Twenty-four hours after AMPT injection, the concentration of DA still did not recover to the initial level. However, there were slight differences in DA levels in the striatum of NET-KO and WT mice after AMPT injection (two-way ANOVA *p* = 0.038, factor “genotype”; the Sidak multiple comparison test revealed no significant differences).

**Figure 2:**
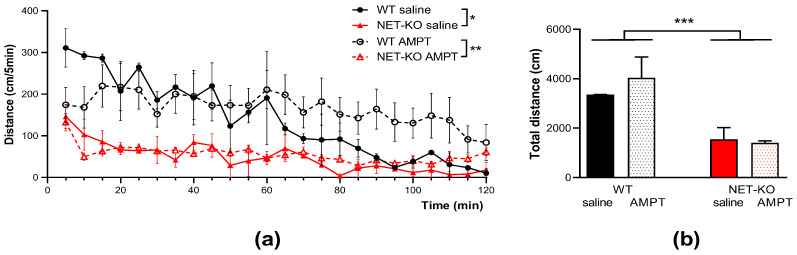
The effect of AMPT on locomotor activity in NET-KO and WT mice (n = 10 per group). (**a**) Dynamics of locomotor activity following administration of AMPT (250 mg/kg) or saline IP; (**b**) The total distance in 2 h following administration of AMPT (250 mg/kg) and saline IP; *—*p* < 0.05; **—*p* < 0.01; ***—*p* < 0.001; two-way ANOVA.

After confirming a significant decrease in NE levels in areas where it is released from norepinephrine terminals, we assessed changes in motor function under the influence of AMPT. The results of comparing the locomotor activity of NET-KO and WT mice after saline administration (Figure 2) were consistent with the results described earlier in the literature for characterizing the NET-KO phenotype [48]. Knockout mice were significantly less active than WT mice. They traveled significantly shorter distances for every five minutes spent in the experimental boxes (Figure 2a; *p* = 0.015, factor “genotype”, two-way ANOVA). A comparison of the distance traveled by KO and WT mice over the entire two-hour recording period also revealed significant differences between the two groups (*p* < 0.001; one-way ANOVA).

However, AMPT injection did not affect this parameter in either NET-KO (*p* = 0.97; one-way ANOVA) or control mice (*p* = 0.32; one-way ANOVA) (see Figure 2b). Thus, intraperitoneal administration of AMPT did not lead to statistically significant differences between the genotypes, but the difference found when comparing the locomotor activity of WT and NET-KO mice persisted even after administration of AMPT (factor “genotype”; *p* = 0.005, two-way ANOVA).

**Figure 3:**
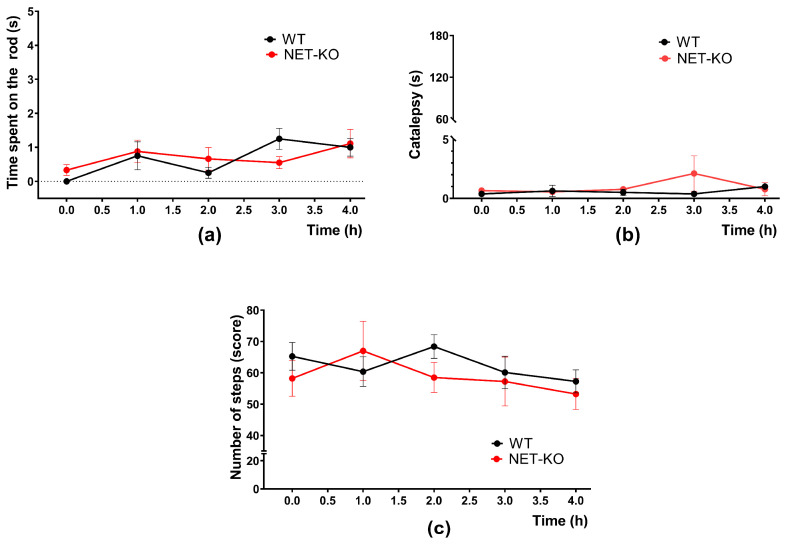
The effect of AMPT on the motor activity of WT (n = 8) and NET-KO (n = 9) mice tested in a group of behavioral tests. (**a**) Grasping test of muscle rigidity. (**b**) Catalepsy test. (**c**) Akinesia test.

Since we found no significant changes in motor activity, we performed a battery of tests to see if there were any problems with movement control after AMPT administration. We used the same tests that revealed the “akinetic” phenotype of DAT-KO mice after AMPT administration [46]: a test for muscle rigidity (Figure 3a), catalepsy (Figure 3b), and akinesia (Figure 3c). We did not detect any signs of akinesia, catalepsy, or muscle rigidity in either WT or NET-KO mice (factor “genotype”, not significant, two-way ANOVA).

**Figure 4:**
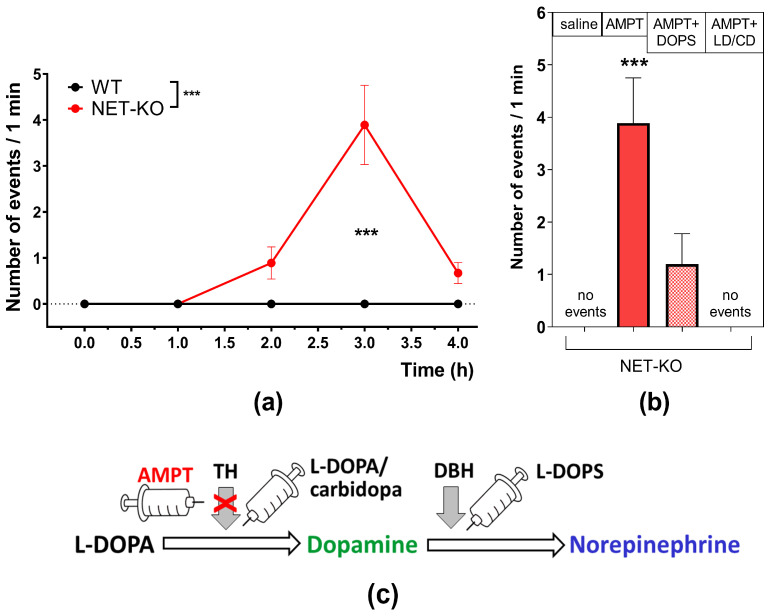
The effect of AMPT administration on the hindlimb body extension test in WT (n = 8) and NET-KO (n = 9) mice: (**a**) the dynamic of the hindlimb body extension test after AMPT administration; *** *p* < 0.001, two-way ANOVA with Sidak’s multiple comparisons test; (**b**) comparative effect of AMPT, L-DOPS, and L-DOPA/carbidopa (LD/CD) administration; *** *p* < 0.001; one-way ANOVA with Dunnett’s multiple comparisons test; and (**c**) the drug administration schedule includes a dopamine synthesis blocker (AMPT), dopamine (LD/CD), and norepinephrine (L-DOPS) precursors.

However, administration of AMPT caused NET-KO mice, but not WT, to demonstrate extension of the hindlimbs (Figure 4a). We assessed the number of abnormal limb extensions observed exclusively in knockout animals after AMPT injection (factor “genotype”; *p* < 0.001, two-way ANOVA). Hind extension started to show 2 h after AMPT injection (not significant, *p* = 0.20, two-way ANOVA with Sidak’s multiple comparisons test) and reached its maximum at 3 h (*p* < 0.001, two-way ANOVA with Sidak’s multiple comparisons test). No such effect was observed in WT mice.

To check if we could reverse that kind of behavior, we performed intraperitoneal injections of the NE precursor L-DOPS (5 mg/kg) and the catecholamine precursor L-DOPA (50 mg/kg), in combination with carbidopa (20 mg/kg), 2 h after AMPT (Figure 4b). We observed that, compared to saline, administration of AMPT significantly increased the manifestation of limb extension in NET-KO mice (*p* < 0.001, one-way ANOVA with Dunnett’s multiple comparisons test). The administration of L-DOPS reduced limb extension behavior in NET-KO mice (*p* = 0.008), while the administration of L-DOPA/carbidopa eliminated this behavior (*p* = 0.0002; one-way ANOVA with Dunnett’s multiple comparisons test), with no significant difference compared to saline administration (Figure 4b,c).

## 3. Discussion

The aim of this work was to test whether a lack of norepinephrine in NET-KO mice caused by the administration of a tyrosine hydroxylase enzyme blocker, AMPT, could provoke movement disorders characteristic of PD. The study was conducted using transgenic mice that lacked the norepinephrine transporter (NET-KO mice). This led to an excessive accumulation of norepinephrine (NE) in the extracellular space in regions where the NET is normally located, such as the prefrontal cortex (PFC), hippocampus, and cerebellum [48]. The preference for using a transgenic line instead of pharmacological inhibition of the norepinephrine transporter was associated with the abundance of the described non-targeted effects and the non-specificity of agents acting on NET, which makes it difficult to separate the altered effects of dopaminergic and norepinephrine effects. The genetic deletion of the NET in mice is an alternative option for studying the specific effects of dysfunction within the norepinephrine system [52,53,54].

As previously shown, the content of NE in the tissues of NET-KO is significantly lower than in WT mice [48], and our study confirmed that. Intraperitoneal administration of AMPT to NET-KO mice significantly reduced the concentration of NE in the tissues of the frontal cortex. However, due to the fact that WT mice have a mechanism of NE reuptake, temporary inhibition of TH is compensated by circulating NE, due to which a decrease in the concentration of NE tissue is not critical.

NET is a transporter that is mainly responsible for the reuptake of NE from the synaptic cleft. In NET-KO mice, the lack of a mechanism for NE reuptake leads to all of the released NE accumulating in the extracellular space, being unable to return to presynaptic neurons. To compensate for the increased action of NE in the synaptic cleft, the total amount of NE in brain tissue is reduced by more than 2.5 times compared to WT mice [48]. In the absence of the reuptake mechanism, all NE in the neurons of NET-KO mice is synthesized de novo. Therefore, temporarily inhibiting tyrosine hydroxylase can lead to the animals being almost completely depleted of NE. In our experiments with NET-KO mice, the effect of AMPT is reversible. Eight hours after injection, we observe an increase in norepinephrine concentration (see Figure 1a,b).

As previously shown, the basic locomotor activity of NET-KO mice was reduced relative to that of wild-type mice [48], and we obtained a similar result in our work. Administration of AMPT did not change locomotor activity in both wild-type and knockout mice. Additionally, conducting a set of behavioral tests, including the grasping, akinesia, and catalepsy tests, revealed no signs of motor disorders consistent with a number of Parkinsonian symptoms. As we have shown, injection of AMPT lowered the concentration of NE in the frontal cortex almost to zero 1 h after injection in NET-KO mice (Figure 1a). Such a drastic reduction in NE concentration had no effect on locomotor activity and did not lead to akinesia, catalepsy, or changes in grasping (Figure 2). This suggests that an acute decrease in extracellular NE concentration does not affect these types of motor behavior.

An interesting result obtained from the conducted study was the manifestation of hindlimb extensions in NET-KO mice, the maximum development of which was detected at the third hour after administration of AMPT (Figure 4a). This correlates with the period of complete depletion of norepinephrine in the studied tissue (Figure 1a). The administration of both an artificial precursor to norepinephrine, L-DOPA/carbidopa, and a precursor to catecholamines, L-DOPA/carbidopa, resulted in the leveling of this effect (Figure 4b). Similar to the hindlimb extensions shown in our study, motor disorders in dyskinetic behavior were observed by the authors in mice with defects in the noradrenergic system, including DBH-/- mice and mice treated with the DSP4 neurotoxin, which led to lesion of the LC [55]. In particular, there was an increase in abnormal limb scores and severe paw tremors at rest. These symptoms were reduced by the administration of NE.

One of the early symptoms of PD is spasticity of the muscles in the limbs and trunk, which may appear before classic motor symptoms such as tremor or bradykinesia [1]. The abnormal hindlimb extension symptoms observed in NET-KO mice after AMPT administration can be compared to the manifestations of restless legs syndrome (RLS) [56]. The literature contains data on an increased risk of RLS in patients with PD, as well as suggesting a possible link between the two diseases [57]. This syndrome is associated with dysfunction of the DA and NE systems [58]. It may manifest itself in patients during the development of PD or act to predict the onset of PD [59,60].

Striatal dopamine is one of the most important players involved in motor control [61,62,63,64,65,66]. Previously, colleagues used DAT-KO mice to deplete dopamine levels using AMPT (DDD mice). DDD mice exhibit an extremely akinetic phenotype and show symptoms similar to Parkinson’s disease, including akinesia, rigidity, tremor and ptosis [46]. However, observations have shown that a strong stressor may cause a temporary disruption of such a phenotype [46,47]. In addition, a similar model using DAT-KO rats was created [67]. All these studies show the importance of dopamine in the manifestation of motor symptoms of the late stages of PD [47,68]. In NET-KO mice, administration of AMPT led to a decrease in DA level, but the decrease was the same as in WT mice. The DA concentration 2 h after injection of AMPT did not differ between NET-KO and WT. It is not likely that a decrease in DA concentration is the reason for the observed limb extensions, as WT did not show such behavior.

There is an assumption that dyskinesia caused by dopamine replacement therapy in PD patients is an abnormality of synaptic plasticity in dopaminergic neurons, associated with loss of support from the noradrenergic system. α2-adrenergic antagonists improve motor symptoms in patients with PD and may also reduce levodopa-induced dyskinesia [12]. The beta-adrenergic antagonist nadolol inhibits the self-control of neurons over the release of neurotransmitters, thereby increasing the concentration of extracellular norepinephrine. This drug reduces tremor in PD. The artificial precursor to norepinephrine, L-DOPA, improves gait and balance in PD patients [19]. In our study, injection of L-DOPS significantly decreased abnormal limb movements. Interestingly, injection of the DA precursor L-DOPA/carbidopa had an even stronger effect—removing abnormal movements completely. L-DOPA is a precursor of DA and therefore also a precursor of NE, as NE is synthesized from DA. It is possible that such action is due to the fact that L-DOPA can increase the concentration of both DA and NE. A manifestation of abnormal hind extension is relevant to both DA and NE systems. It is possible that a decrease in DA concentration that was not significant in NET-KO mice, due to the almost full absence of NE and its action on the DA system, leads to abnormal limb movement.

In our study, an injection of L-DOPS significantly reduced the abnormal limb movements induced by AMPT, whereas an injection of the dopamine precursor L-DOPA/carbidopa eliminated them entirely. This may be due to reduced norepinephrine levels in the prefrontal cortex and other parts of the CNS, including the hippocampus and cerebellum, as has been demonstrated in previous studies [48]. However, the early behavioral response (one hour after AMPT administration) may result from decreased NA levels in peripheral tissues, reflecting the peripheral effects of norepinephrine. It can be hypothesized that the observed events may be primarily related to changes in peripheral NE levels. Further studies and analysis of NE level fluctuations in both other CNS structures and peripheral tissues are needed to elucidate the mechanism underlying the observed behavioral abnormalities.

NE plays a number of roles in ensuring normal DA neurotransmission. NE neurons of the LC widely innervate DA neurons in the midbrain, including the SN and VTA [69]. Key components of the NE system can be found in DA neurons in these regions, including alpha- and beta-adrenergic receptors, NET, and DBH. Stimulation of the LC leads to activation of DA neurons, which can be modulated by antagonists of various types of adrenoreceptors [70,71,72,73,74]. For example, presynaptic alpha2-adrenergic receptors negatively regulate the release of DA from DA terminals [74,75]. Damage or depletion of NE can reduce basal or amphetamine-induced DA release in the striatum [76]. As found in our study, the partial decrease in tissue dopamine concentration in the striatum after AMPT administration may be associated with a corresponding acute depletion of NE. One of the possible reasons for close interaction between the two neurotransmitter systems is overlapping signaling pathways. For example, alpha2-adrenergic receptors have two opposing effects on dopamine signal transmission: inhibiting D1 receptor signaling in striatonigral neurons and enhancing D2 receptor signaling in striatopallidal neurons [75]. Alpha2 adrenergic receptors associated with Gi interfere with the transmission of signals through Gs/olf-related D1 receptors, while, at the same time, alpha2 adrenoreceptors contribute to D2 receptor signaling, as D2 receptors are also associated with Gi proteins [75,77,78].

**Figure 5:**
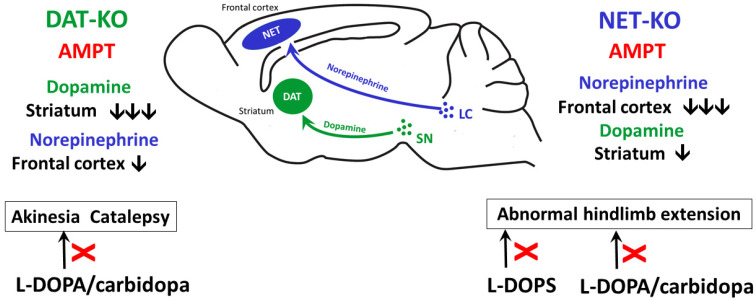
A comparison of the effects of AMPT administration on DAT-KO and NET-KO mice. The arrows indicate a decrease in DA and NE tissue levels in the corresponding brain structures. The box shows the types of motor disorders observed. L-DOPA/carbidopa is a DA synthesis precursor, and L-DOPA is an NE synthesis precursor from DA.

So, the preliminary results suggest that the DA synthesis inhibitor AMPT disrupts both the dopaminergic and noradrenergic systems. In DAT-KO mice, AMPT sharply reduces DA levels in the striatum but not NE levels in the frontal cortex, leading to pronounced akinesia and the development of cataleptic states lasting several hours after injection [47]. These manifestations can be compensated for by administering the DA precursor L-DOPA (Figure 5).

In NET-KO mice, AMPT causes an equally sharp decrease in NE levels in the frontal cortex without such pronounced akinesia. However, the abnormal hindlimb extension observed after AMPT against the background of changes in NE levels in the cortex and DA levels in the striatum of knockout mice indicates the involvement of the NE system in the regulation of dopamine transmission. This is confirmed by an experiment in which the number of abnormal hindlimb extensions is significantly reduced by using the DA precursor L-DOPA and/or the NE precursor L-DOPS (Figure 5).

The data presented here do not claim to provide a description of the interaction between the dopaminergic and noradrenergic systems in the brain or of their mutual influence. To clarify the role of NE in the regulation of DA signaling, it would be interesting to investigate whether NE levels change in DAT-KO animals with hypodopaminergy in the striatum (Figure 5). This would help us to better understand the possible role of NE in the development of the early symptoms of PD.

This study has several limitations. First, our focus was on the prefrontal cortex, given its key role in integrating DA and NE projections. Direct analysis of NE levels in the LC was not possible due to the structure’s extremely small size in mice, which would not have allowed sufficient material to be obtained for analysis. Secondly, the obtained data do not enable us to distinguish between the contributions of peripheral and central factors to the effects of NE depletion. Consequently, it is not yet possible to determine conclusively whether the observed changes are due to alterations in the CNS, changes in peripheral NE levels, or both. Finally, further research is needed into potential behavioral abnormalities caused by the blockade of DA and NE synthesis, since the data obtained in our study are limited to a narrow range of behavioral measures.

## 4. Materials and Methods

### 4.1. Animals

In our experiments, we used 100 adult mice: NET knockout (NET-KO; n = 50) and wild type (WT; n = 50) males, aged 3–4 months. The NET knockout mouse line was generated by the laboratory of Dr. Marc Caron at Duke University, Durham, NC, USA. These lines were originally generated using 129SvJ mouse strain ES cells (the gene for the mouse NET was disrupted in embryonic stem cells by homologous recombination through inactivation of exon 2) and have been back-crossed for >10 generations onto the C57BL/6J strain background [48]. Mice were group-housed on a 12 h/12 h light/dark cycle and given ad libitum access to food and water. All tests were performed during the light period of the light/dark cycle. All experiments were conducted with an approved protocol from the Duke Institutional Animal Care and Use Committee (A236-04-08, 20 December 2006) and followed the National Institutes of Health guidelines. The brain tissue samples obtained were examined at the Molecular and Cellular Technologies Research Center at Saint Petersburg State University in accordance with the Ethics Committee protocol No. 131-03-6; approval date: 25 April 2025.

### 4.2. Drugs

AMPT (α-methyl-DL-tyrosine methyl ester hydrochloride); Sigma-Aldrich (St. Louis, MO, USA); CAS#: 7361-31-1) was used as a tyrosine hydroxylase inhibitor. AMPT (250 mg/kg) was dissolved in saline (0.9% NaCl) and was administered intraperitoneally (IP) as a single dose. L-DOPS (L-dihydroxyphenylserine; Sigma-Aldrich; CAS#: 23651-95-8) was used as an artificial precursor of norepinephrine (5 mg/kg) and was administered intraperitoneally (IP) as a single dose [79]. L-DOPA (3,4-Dihydroxy-L-phenylalanine; Sigma-Aldrich; CAS#: 59-92-7) was used as a precursor of catecholamines (50 mg/kg), and carbidopa (Sigma-Aldrich; CAS#:38821-49-7) was used as a blocking agent of the peripheral metabolism of L-DOPA (20 mg/kg). Both L-DOPA and carbidopa were administered intraperitoneally (IP) as a single dose.

### 4.3. Behavioral Tests

Behavioral experiments were conducted using 10 knockout and 10 WT mice. To minimize potential confounding factors, all experiments were conducted at the same time of day, with the participant supervising the experimental design. All behavioral experiments were performed between 10:00 a.m. and 5:00 p.m. The experiments were conducted in a specially designed room to maintain a quiet environment. Before the behavioral experiment began, the mice were acclimatized to the room for an hour.

During the entire study, the mice were under visual control before and after the administration of AMPT. Intraperitoneal injections of AMPT were administered once.

The time of the AMPT administration in each graph in the Results chapter is marked as the zero point. The behavioral test panel was used in a similar way to the study of the akinetic phenotype in DDD mice [46].

*Locomotor activity* was measured in an Omnitech CCDigiscan (Accuscan Instruments, Columbus, OH, USA) activity monitor under bright illumination. All behavioral experiments were performed between 10:00 AM and 5:00 PM. Activity was measured at 5 min intervals. To evaluate the effects of drugs on locomotor activity, mice were placed in monitor chambers (20 × 20 cm) after treatment with AMPT (250 mg/kg IP). The analyzed parameters were the dynamics of locomotor activity and the total distance in 120 min.

*Akinesia test (number of steps)*: A mouse was held so that it was standing on forelimbs only and moving on its own. The number of steps taken with both forelimbs was recorded during a 30-s trial. The assessment of akinesia was conducted immediately following the administration of AMPT and then repeated every hour for a period of four hours.

*Catalepsy test (horizontal wooden bar)*: Catalepsy was determined by placing forelimbs on the horizontal wooden bar (0.8 cm) positioned 4 cm above the surface. The time the animal spent on the bar was recorded (maximum time—3 min). The assessment of the catalepsy was conducted immediately following the administration of AMPT and then repeated every hour for a period of four hours.

*Grasping test of muscle rigidity*: A mouse was suspended by its forelimbs on a metal rod (diameter: 0.25 cm) positioned approximately 20 cm above the table. The time the animal remained on the rod (maximum time—1 min) was noted. The assessment of the muscle rigidity was conducted immediately following the administration of AMPT and then repeated every hour for a period of four hours.

*Hindlimb body extension test*: To evaluate the effects of drugs in the hindlimb body extension test, mice were placed in monitor chambers (20 × 20 cm) after treatment with AMPT (250 mg/kg IP). Abnormal limb extension was considered an event. An “event” was defined as simultaneous hindlimb extension (both limbs) lasting ≥1 s. The number of such events has been calculated. A number of events was counted immediately following the administration of AMPT and then repeated every hour for a period of four hours. In additional tests to evaluate the effect of L-DOPS and L-DOPA/carbidopa on abnormal hindlimb extensions, L-DOPS or L-DOPA/carbidopa were administered 2 h after administration of AMPT. The test was performed one hour after the administration of the drugs.

### 4.4. HPLC Method

Concentration of monoamines in the brain tissue of NET-KO and WT mice after intraperitoneal injection of 250 mg/kg AMPT was measured at several time points. We used n = 5 animals per group at each time point. The total number of mice was 80. Brain tissue samples were obtained after decapitation of the mice by cervical dislocation.

Mice were sacrificed 30 min, 1, 2, 4, 8, 16, or 24 h after injection. The frontal cortex (FC) and striatum were dissected on ice, frozen in liquid nitrogen, and stored at −80 °C. Tissue samples were homogenized in 0.1 M HClO4 containing 100 ng/mL 3,4-dihydroxybenzylamine (DHBA) as an internal standard. Homogenates were centrifuged for 10 min at 10,000× *g*. Supernatants were filtered using a 0.22 μm filter and analyzed for levels of norepinephrine and dopamine using HPLC-EC [46]. Monoamines and metabolites were separated on a microbore reverse-phase column (C-18, 5 μm, 1 × 150 mm, Unijet, Bioanalytical Systems Inc. (BASi), West Lafayette, IA, USA) with a mobile phase consisting of 0.03 M citrate-phosphate buffer with 2.1 mM octyl sodium sulfate, 0.1 mM EDTA, 10 mM NaCl, and 17% methanol (pH 3.6) at a flow rate of 90 μL/min and detected by a 3 mm glass carbon electrode (Unijet, BAS) set at 0.8 V. The accuracy of labeling of the brain tissue sample tubes was carefully monitored. When performing HPLC analysis, the participant was blinded to the animal’s genotype.

### 4.5. Data Analysis

Statistical analysis was performed with GraphPad Prism 8.2.1 (GraphPad Software, Inc., San Diego, CA, USA). The data are presented as mean ± SEM. All data were tested for normal distribution in order to select the most appropriate statistical analysis method. If the data did not follow a normal distribution, a non-parametric analysis method was selected instead. We used two-way analysis of variance (two-way ANOVA followed by Sidak’s multiple comparison test) to analyze the time factor, the genotype factor (NET-KO or WT), and the treatment factor (saline or AMPT administration). One-way analysis of variance (one-way ANOVA followed by Dunnett’s multiple comparison) was used to compare the effect of drugs on the behavior of NET-KO mice in the hindlimb body extension test.

## 5. Conclusions

In conclusion, substantial NE reduction in the frontal cortex—a key region for DA/NE interactions—following AMPT administration in NET-KO mice (NDN mice) did not lead to the development of typical motor abnormalities resembling Parkinsonian symptoms. This suggests that isolated noradrenergic system dysfunction does not lead to the pronounced motor deficits associated with Parkinson’s disease, as demonstrated in DAT-KO mice following dopaminergic disruption. However, the manifestation of abnormal hindlimb extension in NET-KO mice, peaking during the period of maximal norepinephrine depletion, indicates a specific role for norepinephrine in the regulation of postural tone and warrants further investigation. The fact that this symptom is relieved by both L-DOPA, a precursor to dopamine synthesis, and L-DOPS, an artificial precursor to norepinephrine synthesis, suggests that both DA and NE are involved in regulation. These findings highlight the importance of targeted manipulation of individual neurotransmitter systems to dissect their distinct contributions to the pathogenesis of neurodegenerative disorders. Using DAT-KO and NET-KO mutants as animal models may enable us to construct a mechanism of interaction between the dopaminergic and noradrenergic neurotransmitter systems by influencing catecholamine levels. Further research into the potentially significant role of NE in regulating dopamine neurotransmission disorders observed in DAT-KO animals would be interesting. This could form the basis for developing new therapeutic strategies aimed at modulating noradrenergic transmission.

## Data Availability

The original contributions presented in this study are included in the article. Further inquiries can be directed to the corresponding author.

## Abbreviations

The following abbreviations are used in this manuscript:

PD	Parkinson’s disease
DA	Dopamine
SN	Substantia nigra
LC	Locus coeruleus
NE	Norepinephrine
BDNF	brain-derived neurotrophic factor
FGF-2	fibroblast growth factor-2
NGF	nerve growth factor
MPTP	1-methyl-4-phenyl-1,2,3,6-tetrahydropyridine
NET	norepinephrine transporter
LPS	lipopolysaccharide
VMAT-2	vesicular monoamine transporter-2
TH	tyrosine hydroxylase
DBH	dopamine-beta-hydroxylase
L-DOPS	Droxidropa
LD/CD	L-DOPA/carbidopa
DDD	Dopamine deficiency in DAT-KO mice
NDN	Norepinephrine deficiency in NET-KO mice
